# Deep Brain Stimulation of Medial Dorsal and Ventral Anterior Nucleus of the Thalamus in OCD: A Retrospective Case Series

**DOI:** 10.1371/journal.pone.0160750

**Published:** 2016-08-09

**Authors:** Mohammad Maarouf, Clemens Neudorfer, Faycal El Majdoub, Doris Lenartz, Jens Kuhn, Volker Sturm

**Affiliations:** 1 Department of Stereotaxy and Functional Neurosurgery, Cologne-Merheim Medical Center (CMMC), University of Witten/Herdecke, Cologne, Germany; 2 Department of Psychiatry, Psychotherapy and Psychosomatic Medicine, Johanniter Hospital Oberhausen, Oberhausen, Germany; 3 Department of Neurosurgery, University Hospital of Würzburg, Würzburg, Germany; National Institue on Drug Abuse, UNITED STATES

## Abstract

**Background:**

The current notion that cortico-striato-thalamo-cortical circuits are involved in the pathophysiology of obsessive-compulsive disorder (OCD) has instigated the search for the most suitable target for deep brain stimulation (DBS). However, despite extensive research, uncertainty about the ideal target remains with many structures being underexplored. The aim of this report is to address a new target for DBS, the medial dorsal (MD) and the ventral anterior (VA) nucleus of the thalamus, which has thus far received little attention in the treatment of OCD.

**Methods:**

In this retrospective trial, four patients (three female, one male) aged 31–48 years, suffering from therapy-refractory OCD underwent high-frequency DBS of the MD and VA. In two patients (de novo group) the thalamus was chosen as a primary target for DBS, whereas in two patients (rescue DBS group) lead implantation was performed in a rescue DBS attempt following unsuccessful primary stimulation.

**Results:**

Continuous thalamic stimulation yielded no significant improvement in OCD symptom severity. Over the course of thalamic DBS symptoms improved in only one patient who showed “partial response” on the Yale-Brown Obsessive Compulsive (Y-BOCS) Scale. Beck Depression Inventory scores dropped by around 46% in the de novo group; anxiety symptoms improved by up to 34%. In the de novo DBS group no effect of DBS on anxiety and mood was observable.

**Conclusion:**

MD/VA-DBS yielded no adequate alleviation of therapy-refractory OCD, the overall strategy in targeting MD/VA as described in this paper can thus not be recommended in DBS for OCD. The magnocellular portion of MD (MDMC), however, might prove a promising target in the treatment of mood related and anxiety disorders.

## Introduction

Obsessive Compulsive Disorder (OCD) is a complex neuropsychiatric disorder whose main features involve persistent, intrusive thoughts (obsessions) and repetitive, ritualistic behaviors aiming to neutralize the distress (compulsions). OCD has severe impact on an individual’s occupational, academic and personal life causing misery and reduced quality of life [1]. Cognitive-behavioral therapy and pharmacotherapy with serotonin reuptake inhibitors and clomipramine have proven effective treatment options in most patients. However, 20–40% of OCD patients show little or no symptom relief to conventional treatment and remain severely affected [2]. Since the first study published by Nuttin et al. in 1999 [3], deep brain stimulation (DBS) has increasingly gained importance as a treatment option in the field of therapy-refractory Obsessive-Compulsive Disorder (OCD). However, the most effective anatomical target for stimulation remains controversially discussed. Recent publications favored the investigation of the internal capsule/ ventral striatum (IC/VS) [3–15] and the nucleus accumbens (NA) [16–25]. Targets such as the subthalamic nucleus (STh) [26,27] and the thalamus [28–30] are underexplored. In an attempt to get a deeper understanding about the efficacy of DBS in the thalamus, we present four patients who underwent lead implantation in the medial dorsal nucleus (MD) and the ventral anterior nucleus (VA) of the thalamus. In two patients the thalamus was chosen as a primary target, whereas in two patients thalamic lead implantation was performed in a rescue DBS attempt following primary stimulation in the NA. In this retrospective study we report the results obtained from our patients during follow up visits.

## Materials and Methods

### Patient selection

Between January 2001 and January 2012, 35 patients suffering from severe, therapy-refractory OCD underwent DBS treatment in the NA at the Department of Stereotactic and Functional Neurosurgery, University of Cologne. Patients who didn’t show any symptom improvement over the course of NA-DBS and remained severely affected by OCD were offered lead replacement and additional lead implantation. Over the course, two patients who did not respond to primary NA stimulation consented to a rescue DBS procedure. Neuroanatomical and pathophysiological considerations (see Rationale for Thalamic DBS) prompted us to select MD/ VA as the most promising target in both cases. In two patients (de novo group), the conventional approach of NA/IC stimulation was abandoned due to distinct depressive symptoms that could be objectified during preoperative assessment (Table 1). The aim in this group was to alleviate both OCD and depressive symptoms employing MD and VA as targets for DBS. Before surgery, every patient was examined and validated by a multidisciplinary team of specialists consisting of neurosurgeons, neurologists, psychiatrists and neuropsychologists. Prior to performing DBS in each patient, the Ethics Committee of the Medical Faculty of the University of Cologne was informed about the extended access-trial. No separate ethics application and statement by the ethical committee for this retrospective study were required. This study has been evaluated in accordance with German data protection legislation (German Data Protection Legislation English Version available as S1 Appendix). This, in particular, means that the results of the study have been obtained in a completely anonymous manner. The authors MM, CN, FE, DL, JK and VS had contact with patients and access to patients’ data during medical treatment and follow-up evaluations. For all kinds of treatment done at the Department of Stereotactic and Functional Neurosurgery Cologne it is mandatory to obtain written informed consent of patients scheduled for treatment. In case of minors, this consent is granted either by their parents or by a court-approved caregiver.

**Table 1:** Demographic data and clinical characteristics at the time of surgery.

Patient No.	Age at MD/VA-DBS (a)	Time of Onset	Duration of OCD (a)	Comorbidity	Suicide attempts	Obsessions	Compulsions	Drug Therapy	Previous Drug Trials	Previous CBT Trials
1	42	Childhood	38	Recurrent depressive disorder, Borderline personality disorder, Bulimia nervosa	0	Fear of contamination	Washing, ordering, counting	Fluoxetine, Diazepam, Lorazepam, Pregabalin	7	12
2	36	Adolescence	19	None	0	Fear of contamination, inappropriate sexual and blasphemous thoughts, fear of misstating facts	Washing, cleaning	Ziprasidone, Fluoxetine	8	9
3	48	Adulthood	17	Posttraumatic stress disorder, Dissociative disorder, Recurrent depressive disorder, Histronic and borderline personality disorder	3	Fear of being touched and contaminated	Washing, cleaning	Sertraline, Quetiapine, Clonazepam	15	14
4	31	Childhood	20	None	0	Fear of the future, interpreting numbers and colors, calculating rituals	Checking, repeatedly touching objects, avoiding to step on stains on the floor	Quetiapine, Duloxetine, Palliperidone	12	11

Patients were eligible for DBS if they were between 21 and 65 years of age and suffered from primary OCD, verified with the Structured Clinical Interview for DSM-IV, German Version. Severity of illness, as assessed with the Yale-Brown Obsessive Compulsive Scale (Y-BOCS) must have been 25 or higher and patients had to attest disease duration of at least 5 years with less than 35% symptom reduction following pharmacologic therapy and cognitive behavioral therapy (CBT) with exposure and response prevention comprising at least 1 CBT trial for a minimum of 20 sessions, each ranging from 60–120 minutes. Pharmacotherapy involved treatment with a selective serotonin reuptake inhibitor (SSRI) for at least 10 weeks, an additional treatment regimen using a different SSRI or clomipramine over a period of 10 weeks and augmented therapy administering an atypical antipsychotic, lithium or buspirone for 10 weeks. Table 1 summarizes clinical characteristics and demographic data of the patients at the time of surgery.

### Rationale for thalamic DBS

**Fig 1:**
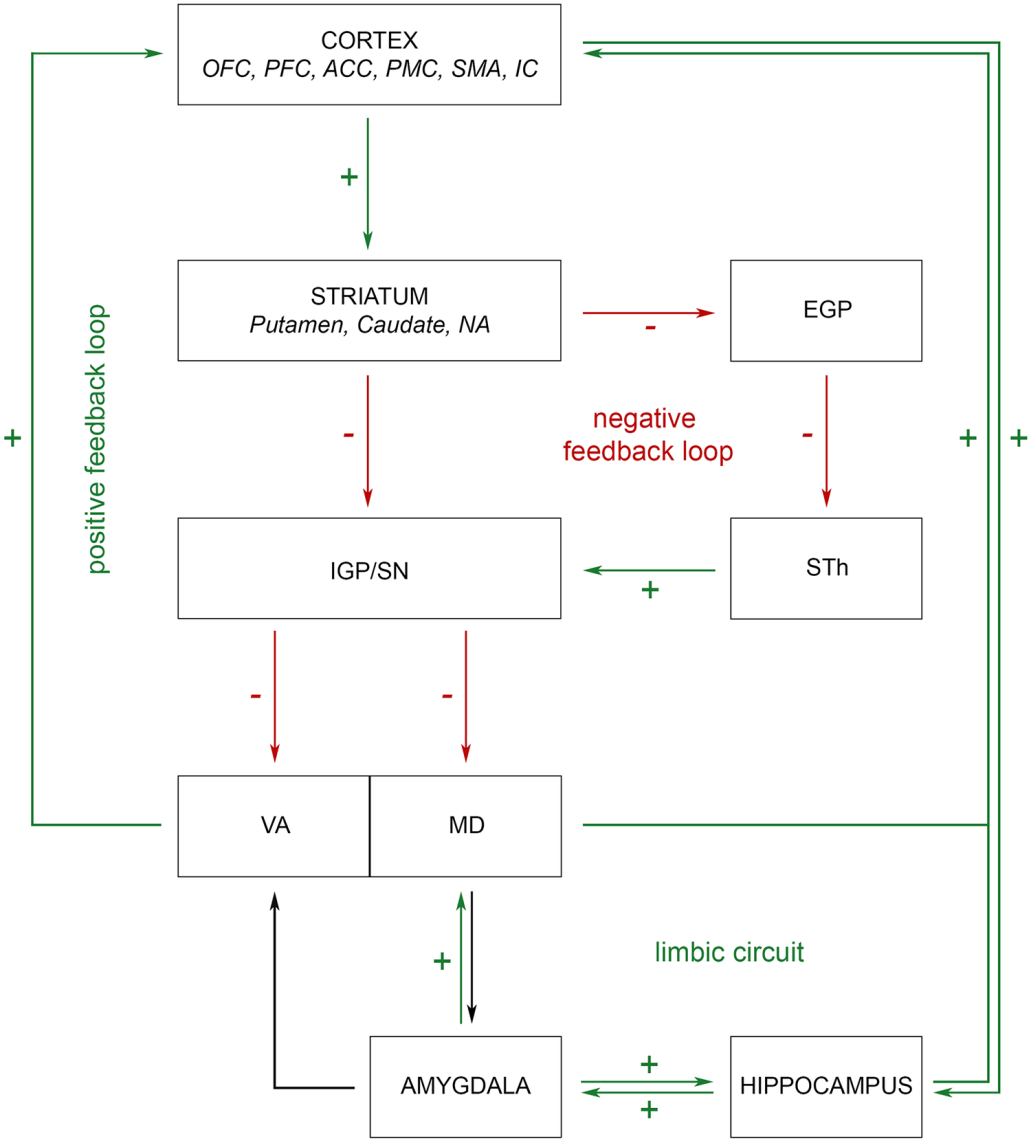
Schematic of thalamic involvement in OCD pathophysiology. MD and VA are embedded within the “direct” positive and “indirect” negative feedback loop of the CSTC circuit and are under striatal influence. Imbalance between direct and indirect pathways results in increased CSTC activity in OCD. Amygdaloid afferents link MD and VA with limbic circuits and enable thalamic processing of emotional stimuli. Increased thalamic output in OCD alters mood and anxiety components. OFC, orbitofrontal cortex; PFC, prefrontal cortex; ACC, anterior cingulate cortex; PMC, premotor cortex; SMA, supplementary motor area; IC, insular cortex; NA, nucleus accumbens; EGP, external globus pallidus; STh, subthalamic nucleus; IGP, internal globus pallidus; SN, substantia nigra; VA, ventral anterior thalamic nucleus; MD, medial dorsal thalamic nucleus.

The involvement of cortical and subcortical structures in the pathophysiology of OCD was discovered during the era of ablative surgery. Surgical disruption of aberrant circuitry by means of anterior capsulotomy [31–34], subcaudate tractotomy [35,36], limbic leucotomy [37,38] and cingulotomy [39–41] aimed for alleviation of obsessions, compulsions and comorbid symptoms. Hence, ablative procedures, along with lesional studies and, more recently, functional imaging modalities built the foundation of our current understanding of the cortico-striato-thalamo-cortical (CSTC) -based model of OCD. The circuit comprises cortical structures such as the orbitofrontal cortex (OFC), prefrontal cortex (PFC) and the anterior cingulate cortex (ACC), the basal ganglia: striatum, pallidum, nucleus accumbens (NA), STh and substantia nigra (SN), the thalamus and limbic components (amygdala, hippocampus) [42–46]. These neuroanatomical structures are interconnected forming two antagonistic pathways, a “direct” positive feedback loop and an “indirect” negative feedback loop (Fig 1). Within the direct circuit, cortical projections from the OFC, PFC and ACC modulate target cells in the striatum. Striatal excitation exerts inhibitory effects on downstream targets in the globus pallidus internus (IGP) and substantia nigra pars reticulate (SNR), which ultimately results in increased reciprocal feedback via thalamo-cortical projections. In contrast, activation of the indirect pathway leads to excitation of IGP and SNR through STh disinhibition. Inhibitory nigro/pallido-thalamic projections subsequently decrease thalamic output and induce negative cortical feedback. In healthy controls, direct and indirect pathways are in balance. In OCD, increased activity of the CSTC circuit is observable at rest due to overactivation of the excitatory pathway as well as failure of inhibition in the indirect loop [43,44,47].

Multiple superordinate systems are embedded in the direct and indirect loop of the CSTC circuit: Motor circuit, associative circuit, limbic circuit, dorsal and ventral cognitive circuit. Pathways reside within segregated basal ganglia territories and remain distinct throughout the CSTC loop although there is considerable crosstalk between circuits [4,48]. Among functional circuits, the ventral cognitive network has consistently been implicated in the pathophysiology of OCD featuring nodes within OFC, head of the caudate nucleus and MD. Lack of inhibition within this circuit is believed to result in anxiety provoking thoughts and conditioned fear responses involving repetitive, intrusive movements and complex acts [49–53]. The thalamus constitutes the final subcortical link within the CSTC loop. When uncoupled from inhibitory striatal influence, thalamic projections exert excitatory effects on the cortex and thus are crucially involved in diverse cognitive and executive tasks, such as strategy selection, behavioral flexibility and prospective coding [54]. Several thalamic nuclei are implicated in the pathophysiology of OCD, predominantly the medial dorsal (MD) and the ventral anterior (VA) nucleus [45,46,55,56]:

MD consists of three subnuclei, that is, the medial or magnocellular nucleus (MDMC), the intermediate or parvocellular nucleus (MDPC), and the lateral or paralaminar nucleus (MDPL) [57]. Each subdivision sends projections to specific areas within PFC, OFC, ACC, premotor cortex and insular cortex, which then relay back to the same location in MD. MDMC receives subcortical afferents from IGP/SNR [58,59] that inhibit MD output, whereas projections from the basal amygdala, the magnocellular nuclei of the basal forebrain and the brainstem stimulate thalamic activity [60]. Amygdaloid afferents reach MDMC through the ventral amygdalofugal pathway and the inferior thalamic peduncle (ithp) and are then conveyed to PFC [61]. MDMC therefore is involved in the evaluation, modulation and transmission of emotional processes and affective stimuli. In turn, MD sends fibers to the amygdala and thereby connects with the dorsomedial and lateral hypothalamic nuclei as well as the dorsal nucleus of the vagus nerve [62–64]. In conjunction with auditory and visual signals, MDMC induces vegetative manifestations and agitation upon sensory stimulation [65,66]. Finally, MDMC is involved in memory processing via amygdalohippocampal and orbitofrontal connections [60,67]. MDPC is the largest nucleus in MD and receives input from multiple midbrain structures within the basal ganglia circuit; efferents reach the dorsolateral and dorsomedial PFC [68]. MDPC plays a role in working memory and, together with fibers from MDPL, controls oculomotor activity by establishing connections to the frontal eye field (FEF). Lesions in MD frequently cause cognitive, emotional and behavioral impairment similar to frontal lobe syndrome [69]. Occasionally, memory disruption [70] and loss of insight and judgment [71] can be observed. Bicuculline (GABA-A antagonist) injections into MD in monkey are characterized by motor hypoactivity and induce distinct dysautonomic manifestations [56]. MD thalamotomy has been reported to alleviate schizophrenic symptoms and anxiety [72].

VA is strongly implicated in the CSTC circuit and processes prefrontal associative, sensorimotor and limbic information. Subcortical afferents from IGP reach the lateral portion of VA (VAL), whereas fibers from SNR terminate in the medial portion of VA (VAM). IGP and SNR modulate voluntary motor activity and determine the type of motor output when multiple movement options are available [73]. VAM receives oculomotor components from the superior colliculus [74], while amygdaloid afferents link VAM with the limbic circuit. The latter originate in the basal amygdala and terminate in the nucleus lateralis rostralis, pars medialis of the ventromedial VAM [75,76]. Efferents from VAM and VAL project to the premotor cortex and supplementary motor area [77–79] without overlap. VAM additionally relays to prefrontal cortex, cingulate cortex and FEF [80–82]. Lesions of the anterior thalamus are associated with complex behavioral syndromes [83] and result in memory impairment, deficits in motor planning and sequencing as well as perseveration in memory, thinking, spontaneous speech and executive tasks [84,85]. Bicuculline injections within VA trigger OCD-like behavior in monkey, i.e. repetitive food seeking, excessive and time-consuming grooming and unusual manipulation of objects. Interruption of compulsive tasks results in aggressive and agitated behavior [56].

### Our target

**Fig 2:**
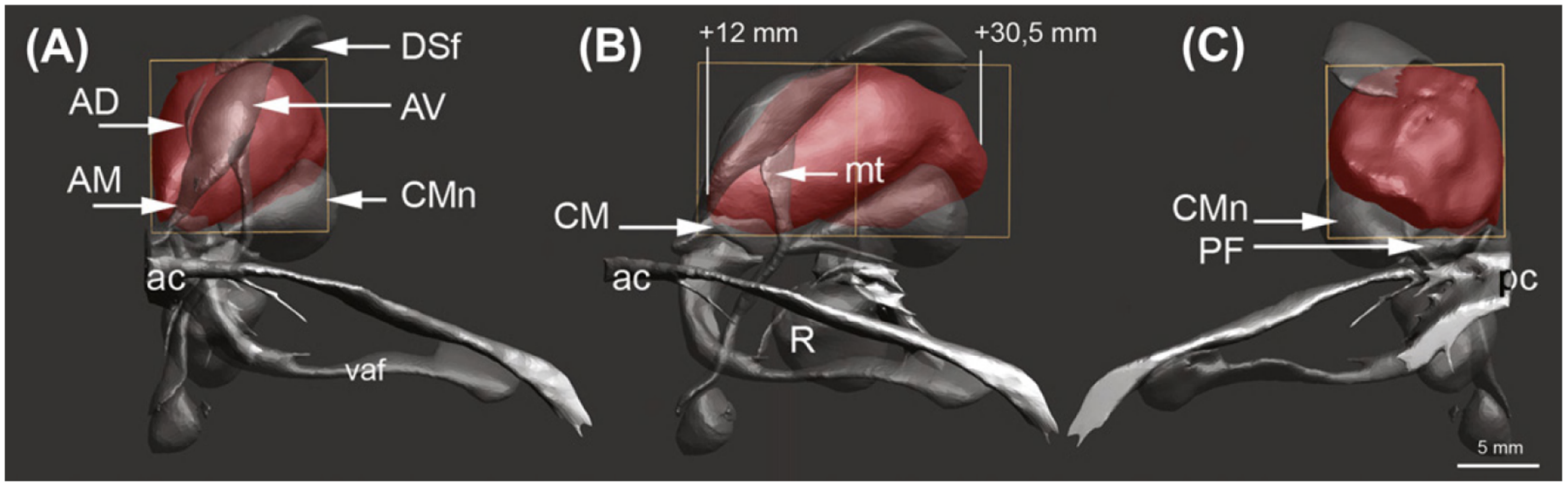
Reconstruction of MD and its boundaries. (A) Coronar section, displaying MD in anterior to posterior-view. (B) Sagittal section of MD. (C) Coronar section of MD in posterior to anterior view. ac, anterior commissure; AV, anteroventral nucleus; CMn, centromedian nucleus; DSf, dorsalis superficialis nucleus; mt, mammillothalamic tract; pc, posterior commissure; vaf, ventral amygdalofugal fibers. Reproduced with permission from Mai JK, Paxinos G (2004): The Human Nervous System, 3rd ed. San Diego Elsevier Academic Press, p 628.

**Fig 3:**
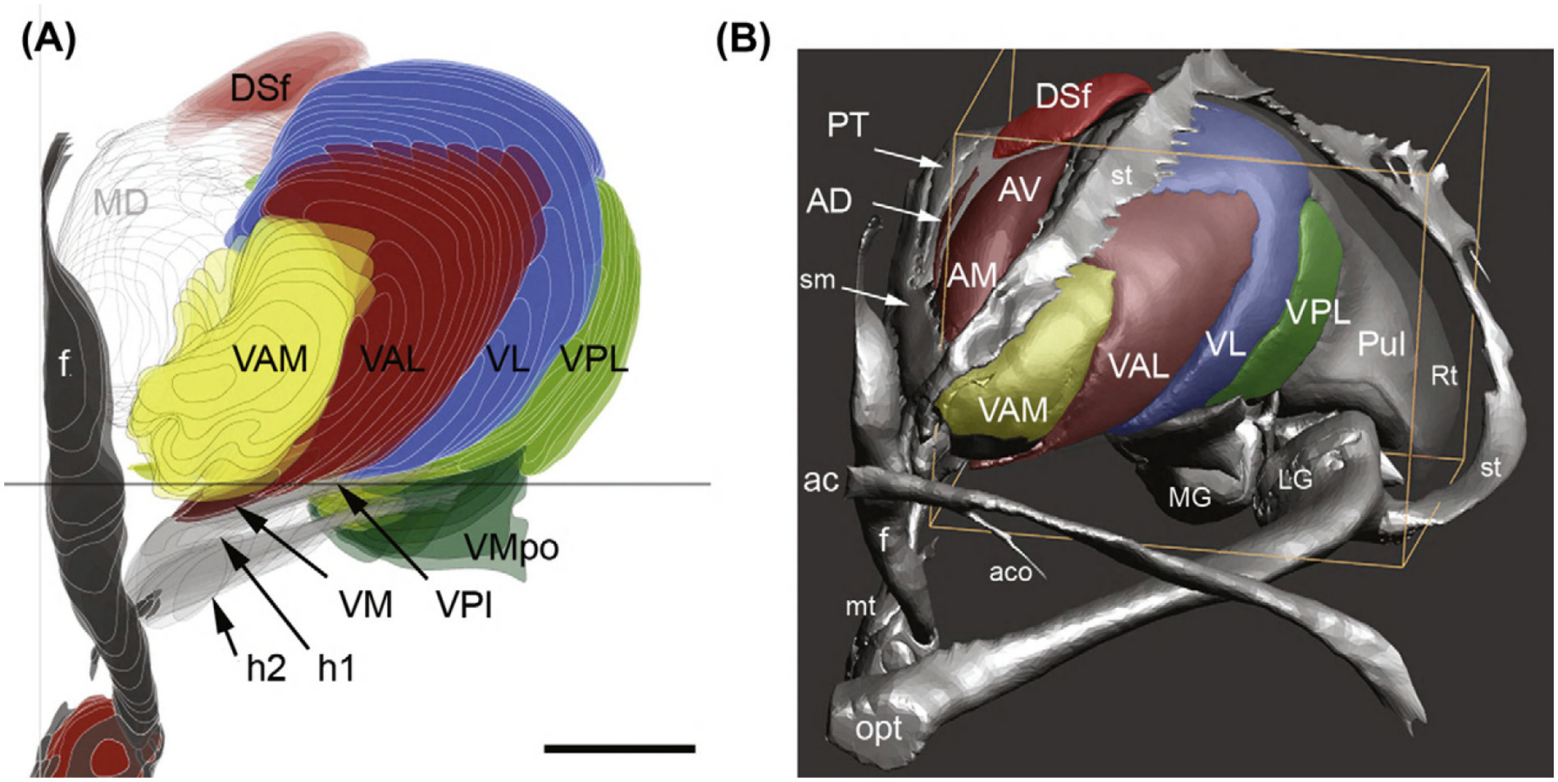
Reconstruction of VA and its surrounding structures. (A) Coronar section of VA in anterior to posterior view. (B) anterolateral surface of VA. ac, anterior commissure; AD, anterior dorsal nucleus; AM, anterior medial nucleus; AV, anterior ventral nucleus; DSf, dorsal superficial nucleus; f, fornix; h1, thalamic fascicle; h2, lenticular fascicle; mt, mammillothalamic tract; Pul, Pulvinar; st, stria terminalis; VAL, ventral anterolateral nucleus; VAM, ventral anteromedial nucleus; VL, ventral lateral nucleus; VM, ventromedial nucleus; VMpo, ventromedial posterior nucleus; VPI, ventroposterior inferior nucleus; VPL, ventral posterolateral nucleus. Reproduced with permission from Mai JK, Paxinos G (2004): The Human Nervous System, 3rd ed. San Diego Elsevier Academic Press, p 637.

According to the Atlas of the Human Brain [86] MD originates on a level with the intrathalamic adhesion, 12.0 mm posterior from the anterior commissure (AC) and extends an estimated 18–20 mm to the level of the habenular commissure (Fig 2). In coronal sections MD features its greatest vertical expansion at 17.2 mm posterior from AC with a length of 13.1 mm. MD’s greatest horizontal diameter amounts to 10.7 mm at 25.2 mm posterior from AC. MD is medially bordered by the paraventricular thalamic nucleus and the third ventricle; ventrally, the ithp advances into MD. The internal medullary lamina (iml) and the intralaminar nuclear group border the lateral side and separate MD from VA. The rostral pole of VA is located 6.7 mm posterior from AC and borders the prereticular zone and the reticular thalamic nucleus (Fig 3). VA expands caudally for 9–10 mm, reaches its greatest extent (16.6 mm) at 12.0 mm and terminates at the ventrolateral nucleus. VAM gets penetrated by the mammillothalamic tract (mt). The lateral margin of VA borders the external medullary lamina; pallidal fibers reach the ventral portion of VA through H1.

### Surgical procedure

**Fig 4:**
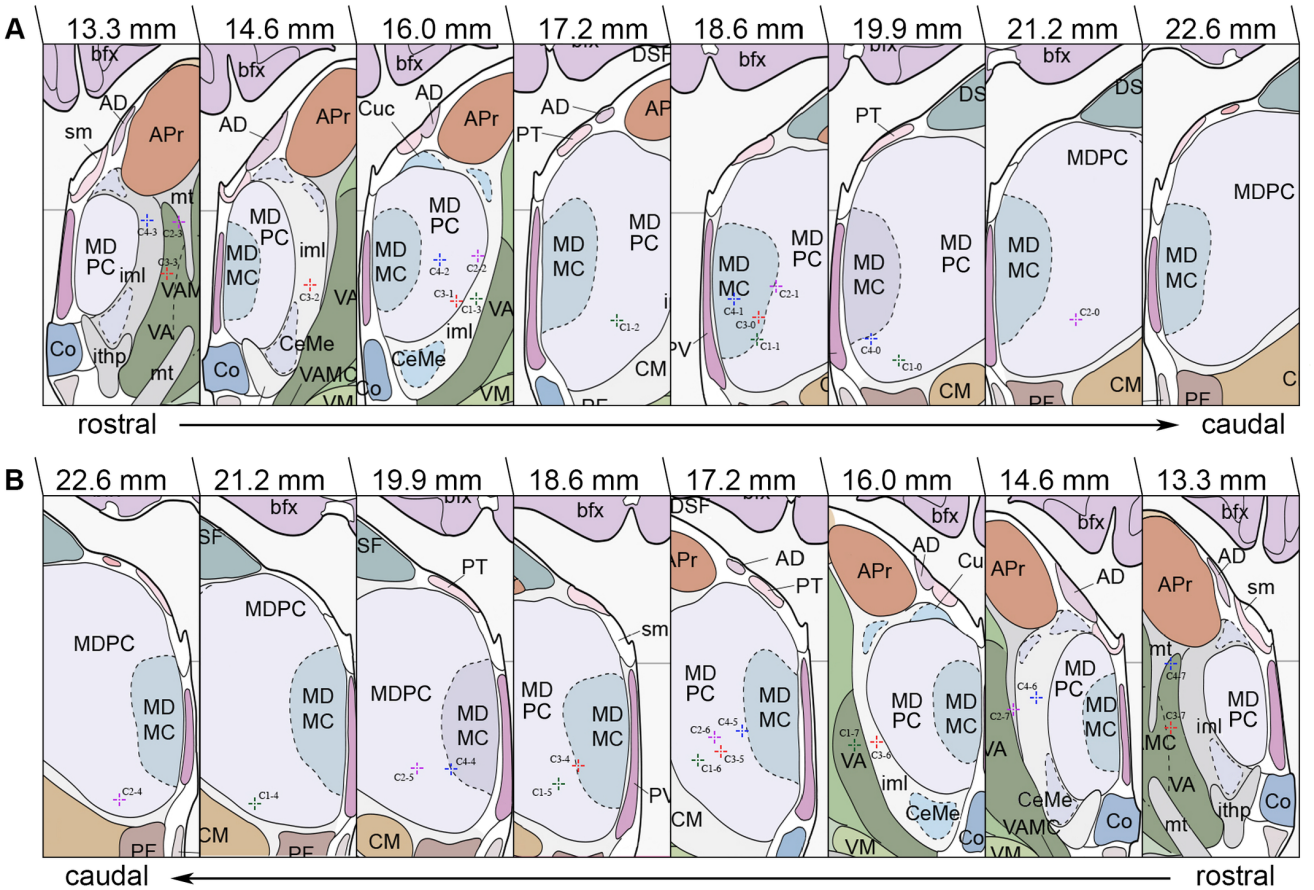
Anatomical localization of MD/VA and DBS lead localization according to the Atlas of the Human Brain. Stereotactic coordinates constitute the centers of active contact points (Case No.-Contact No.) on coronal sections retrieved from postoperative 2D stereotactic X-ray and CCT images. (A) Lead localization within the left hemisphere. (B) Lead placement within the right hemisphere. MDMC, medial dorsal thalamic nucleus, magnocellular part; DSF, dorsal superficial nucleus; bfx, body of fornix; CM, centromedian thalamic nucleus; PF, parafascicular thalamic nucleus; PT, paratenial thalamic nucleus; sm, stria medullaris of thalamus; PV, paraventricular thalamic nucleus; APr, anteroprincipal thalamic nucleus; AD, anterodorsal thalamic nucleus; Cuc, cucullaris nucleus; VA, ventral anterior thalamic nucleus; VM, ventromedial thalamic nucleus; iml, internal medullary lamina of thalamus; CeMe, central medial thalamic nucleus; Co, commissural nucleus; VAMC, ventral anterior thalamic nucleus, magnocellular part; mt, mammillothalamic tract; ithp, inferior thalamic peduncle. Adapted with permission from Mai JK, Paxinos G, Voss T (2007): Atlas of the Human Brain, 3rd ed. San Diego: Elsevier Academic Press.

Prior to surgery frame-based imaging modalities were obtained for stereotactic treatment planning: Magnet Resonance Imaging (MRI) was carried out in axial T1 and T2-weighted sequences while intraoperative cerebral computed tomography (CCT) scans were performed following intravenous administration of contrast agent. After image fusion, target points were determined based on the Atlas of the Human Brain [86] and the surgical trajectory was modified so DBS leads would pass through VA and MD with the lowest contacts (contacts 0 and 4) residing in the mediobasal portion of MDPC. The objective was to place distal contact points within MDPC/MDMC while proximal contact placement was aimed at the transition of MD/VA and in VA, respectively (Fig 4). After burr hole craniostomy and dural incision patients underwent stereotactic-guided lead placement with quadripolar electrodes (Medtronic 3387 in cases 2 and 4 and Medtronic 3389 in cases 1 and 3; Medtronic, Inc., Minneapolis, MN, USA) being implanted bilaterally into the predetermined targets in general anesthesia. To confirm accurate lead positioning 2D stereotactic X-ray procedures and postoperative CCT scans (Philips MX8000 IDT16, Philips Medical Systems, Best, The Netherlands, matrix size 512 x 512, field-of-view 290 mm, slice thickness 1.5 mm, voltage 120 kV, current time product 390 mAs) were obtained. In a subsequent procedure a programmable implantable pulse generator (IPG; Kinetra (case 2), Activa PC (cases 1, 3 and 4), Medtronic Inc., Minneapolis, MN, USA) was placed subcutaneously in an abdominal (case 2) or infraclavicular (cases 1, 3 and 4) pocket and connected to the electrodes using extension wires. Actual stereotactic coordinates were obtained via backward calculation of active contact points using the intercomissural line as a reference. For this purpose, the center of each active contact was determined on postoperative CCT scans using a stereotactic 3D planning software (STP, Stryker-Leibinger, Freiburg, Germany). Coordinates were subsequently adjusted in both anteroposterior and mediolateral plane to match the reference brain as described in the Atlas of the Human Brain. Finally, coordinates were transferred to the Atlas, with each coordinate representing the center of the respective contact (Table 2).

**Table 2:** Coordinates (center of active contacts) of MD/VA electrodes.

	left					right				
Patient No.	Contact number	Contact localization	x	y	z	Contact number	Contact localization	x	y	z
1	0	MDPC	-3.7	20.3	2.4	4	MDPC	5.2	20.6	2.9
	1	MDMC/MDPC	-4.5	18.8	3.5	5	MDPC	5.9	19.0	3.9
	2	MDPC	-5.3	17.2	4.4	6	MDPC	6.7	17.5	5.0
	3	iml	-6.1	15.7	5.5	7	VA	7.4	16.0	5.9
2	0	MDPC	-4.7	20.7	4.5	4	MDPC	4.1	22.6	3.0
	1	MDMC/MDPC	-5.5	18.3	6.2	5	MDPC	5.0	20.1	4.6
	2	MDPC	-6.2	15.9	7.8	6	MDPC	5.9	17.7	6.1
	3	VA/mt	-7.0	13.5	9.4	7	mt	6.7	15.2	7.7
3	0	MDMC	-4.6	18.0	4.7	4	MDMC/MDPC	4.9	18.8	4.8
	1	MDPC	-5.1	16.3	5.4	5	MDPC	5.5	17.1	5.4
	2	iml	-5.7	14.5	6.2	6	iml	6.2	15.3	6.0
	3	VA	-6.4	12.8	6.9	7	VA	6.7	13.5	6.7
4	0	MDMC/MDPC	-2.2	19.9	3.5	4	MDMC/MDPC	3.2	19.3	4.6
	1	MDMC	-3.3	17.9	5.6	5	MDMC/MDPC	4.4	17.1	6.5
	2	MDPC	-4.3	15.9	7.5	6	iml	5.5	15.1	8.2
	3	iml	-5.4	13.9	9.5	7	VA	6.7	13.0	10.0

iml, internal medullary lamina; MDMC, magnocellular nucleus of MD; MDPC, parvocellular nucleus of MD; mt, mammillothalamic tract; VA, ventral anterior thalamic nucleus

### Adjustment of Stimulation Parameters

Determination of optimal stimulation settings in each patient was based on a detailed stimulation protocol obtained during postoperative test stimulation. Consecutive monopolar stimulation of individual contacts was performed initially with a frequency of 130 Hz and a pulse width of 60 μsec. For each active contact, the amplitude was progressively increased to control for acute effects and adverse events. Stimulation was applied sequentially to individual contacts; the trial was discontinued if the patient reported unwanted side effects. During follow-up visits, stimulation parameters were adjusted empirically depending on patients’ response to DBS and neuropsychological scoring. Table 3 gives an overview of stimulation settings used in the immediate postoperative course and during chronic stimulation.

**Table 3:** Stimulation settings following surgery and in the postoperative course.

Patient No.	Time of programming	Stimulation settings
1	Postoperatively	0-, 1-, 2-, 4-, 5-, 6-, c+, 90μs, 130Hz, 4.0V
	1 year follow up	1-, 2-, 5-, 6-, c+, 120μs, 130Hz, 2.5V
	1.5 year follow up	2-, 3-, 6-, 7-, c+, 120μs, 130Hz, 2.7V
2	Postoperatively	0-, 1-, 2-, 4-, 5-, 6-, c+, 90μs, 130Hz, 2.5V
3	Postoperatively	1-, 2-, 5-, 6-, c+, 90μs, 130Hz, 0.5V
	0.5 year follow up	0-, 1-, 2-, 4-, 5-, 6-, c+, 90μs, 130Hz, 3.5V
4	Postoperatively	1-, 2-, 5-, 6-, 90μs, 130Hz, 4.5V
	1 year follow up	1-, 2-, 5-, 6-, 150μs, 130Hz, 3.0V
	2 year follow up	1-, 2-, 5-, 6-, 150μs, 130Hz, 3.0V
	3 year follow up	0-, 1-, 2-, 4-, 5-, 6-, c+, 120μs, 130Hz, 3.5V

### Psychiatric and neuropsychological assessment

After discharge, patients were advised to proceed pharmacologic therapy as normal and under observation by their treating psychiatrists. Modification of stimulation parameters and follow-up evaluation were performed at our outpatient clinic. The primary outcome measure was the change in symptom severity as evaluated by the Y-BOCS. Furthermore an extensive assessment battery was carried out including the Beck Depression Inventory (BDI) [87], State and Trait Inventory (STAI) [88], Modular System of Quality of Life (MSLQ) [89], Global Assessment of Functioning Scale (GAF), Tower of London test (ToL) and Verbal Fluency Examination (VFE). Neuropsychological testing was performed at baseline and after initiating DBS.

## Results

Due to the complexity of the cases reported in this series, each patient is presented as a single case. The first two patients (cases 1 and 2) underwent lead placement in a rescue DBS attempt; cases 3 and 4 received de novo lead implantation (Table 1). Table 2 illustrates the stimulation parameters and target coordinates used for chronic stimulation, and the effect of DBS on Y-BOCS and secondary outcome measures.

Case 1. A 42-year-old single woman with a 38-year history of therapy refractory OCD with mixed obsessional thoughts and acts (ICD-10: F42.2) and without a family history of neuropsychiatric diseases presented for management. Severe OCD symptoms occurred at an early age and she did not recall a specific trigger event. She attended secondary education until the 10^th^ grade; due to the severity of obsessive and compulsive symptoms she was never able to pursue a regular job. Her main obsession is the fear of contamination, which leads her to perform ritualistic washing behaviors. Compulsions are orderliness and counting. The patient’s past medical history revealed recurrent depressive episodes, personality disorder of the borderline type and eating disorder (bulimia nervosa). She underwent psychotherapeutic treatment and was prescribed numerous medications, involving two SSRIs and augmented therapy with quetiapine, different benzodiazepines and anticonvulsants. Therapy hardly yielded any symptom improvements, whereas the patient experienced multiple adverse events during the drug trials and did not show any significant reduction in OCD severity. At admission psychiatric medication included fluoxetine (40–60 mg/d), diazepam (20 mg/d), lorazepam (5.0 mg/d) and pregabalin (600 mg/d).

The patient derived no therapeutic benefit from an initial attempt at stimulation in the right NA and the anterior limb of the internal capsule (ALIC). Therefore, due to treatment refractoriness and in accordance with the patient’s wish, bilateral lead placement into MD/VA was performed 3 years following primary implantation. Scores, however, did not improve after the rescue DBS procedure with thalamic stimulation only (Table 4) and the patient remained disabled and unsatisfied. Given the lack of response to stimulation, the DBS device was eventually explanted.

**Table 4:** Baseline characteristics and outcome of MD/VA stimulation as measured by clinical scales.

Patient No.	Time of follow-up	Y-BOCS	Y-BOCS O	Y-BOCS C	BDI	STAI-X1	STAI-X2	GAF	MSQoL	ToL	VFE
1	Baseline	35	18	17	29	64	64	42	31.9	n/a	n/a
	0.4 months	33	18	15	41	75	68	n/a	22.9	8	37
	3.7 months	31	16	15	38	75	68	42	22.9	1	47
2	Baseline	37	19	18	19	45	52	42	24.6	8	57
	0.3 months	37	19	18	22	45	52	42	24.2	10	52
3	Baseline	32	16	16	42	64	74	41	27.1	12	59
	0.4 months	34	17	17	21	42	73	43	22.1	11	56
	7.5 months	32	15	17	n/a	n/a	n/a	n/a	n/a	n/a	n/a
4	Baseline	35	17	18	41	67	76	41	29.2	n/a	8
	1.7 months	33	16	17	26	63	55	46	52.9	2	16
	13.4 months	29	15	14	24	55	51	43	52.5	10	22
	34.6 months	24	12	12	n/a	n/a	n/a	n/a	n/a	n/a	n/a

BDI, Beck Depression Inventory; GAF, Global Assessment of Functioning; MSQoL, Modular System for Quality of Life; STAI, State (X1) Trait (X2) Anxiety Inventory; ToL, Tower of London Task; VFE, Verbal Fluency Examination; Y-BOCS, Yale-Brown Obsessive Compulsive Scale; Y-BOCS O, Yale-Brown Obsessive Compulsive Scale Obsessions; Y_BOCS C, Yale-Brown Obsessive Compulsive Scale Compulsions

Case 2. A 36-year-old woman, suffering from therapy refractory OCD with mixed obsessional thoughts and acts (ICD-10: F42.2). Onset of disease was at age 17. No certain trigger event could be determined during psychiatric evaluation and family history is negative for neuropsychiatric diseases. After completing lower secondary education, she became a shoe salesman and after being discharged, an assembly line worker. She lives together with her boyfriend. Main obsessions involve her fear of contamination, which result in ritualistic behaviors concerning body hygiene, dressing and using the restroom. Furthermore, she displays intrusive sexual and blasphemous thoughts and impaired speech due to her fear of misstating facts. Past medical treatment consisted of two SSRIs, Clomipramine, as well as diverse antipsychotics and antidepressants. In- and out-patient psychotherapy was performed repeatedly prior to DBS-indication. At admission for MD DBS the patient’s medication involved ziprasidone (80 mg/d), biperiden (4.0 mg/d) and paroxetine (40 mg/d).

Case 2 was initially implanted a lead targeting the right NA and the adjacent anterior limb of the internal capsule. Baseline characteristics and follow-up evaluations are depicted in Tables 1 and 3. Within the first three months after surgery she reported symptom reduction, however, this condition did not last. She developed new symptoms including covering herself with a blanket in a certain way and repeating the process over and over, as well as concerns with symmetry and checking. The only permanent improvement affected the patient’s speech impairment, which occurred less frequently. Five years following primary placement three additional leads were implanted in a rescue DBS attempt. One lead targeted the left NA whereas two leads were placed bilaterally into MD/VA. Initially stimulation was performed in both targets. To validate the efficacy of sole thalamic stimulation, NA DBS was discontinued one month after surgery. Due to persisting symptom severity the thalamic DBS device was turned off after 4 months of continuous stimulation and sole NA stimulation was resumed.

Case 3. A 48-year-old single woman who suffers from OCD with predominantly compulsive acts (ICD-10: F42.1). Onset of OCD was at age 31 and family history is negative for OCD and any other psychiatric diseases. She attended secondary school until the 11^th^ grade and became an educator after dropping out. However, she was not able to pursue her career due to symptom worsening. OCD symptoms first occurred after the patient had undergone endoscopic surgery and mainly consisted of the fear of being touched or contaminated. These obsessive thoughts lead to ritualistic washing behaviors including washing her hands up to 50 times a day and excessive showering lasting up to three hours per session. Because of her fear of contamination, she refused physical examination. Besides the principal diagnosis of OCD the patient’s medical record revealed the following diagnoses: post-traumatic stress disorder, dissociative disorder and recurrent depressive disorder. Furthermore, she fulfilled criteria for histrionic and borderline personality disorder. The patient reported three suicide attempts resulting from family conflicts and sexual abuse. Besides psychotherapy, drug treatment involved three SSRIs, Clomipramine and augmented therapy with various antidepressants, benzodiazepines and anticonvulsants. At admission to our department the patient’s medication consisted of sertraline (250 mg/d), quetiapine (50 mg/d), and clonazepam (2.0 mg/d).

After lead placement into MD/VA, she initially complained about nausea and vertigo as a result of high stimulation parameters. In the days following surgery the patient reported being ‘more outward-oriented’. OCD symptoms however did not improve. Seven months after thalamic DBS, she presented again at our department with no improvement regarding her fear of contamination and worsening of her ritualistic washing behaviors. Moreover, she had developed new symptoms including compulsive gambling, compulsive buying, tiredness and impaired speech. Due to the deterioration of her condition MD leads were explanted after 6 months of continuous stimulation and, in accordance with the patient’s wish, replaced with bilateral leads targeting the NA. Although compulsive gambling and buying compulsions disappeared after lead replacement the patient remained disabled and therapy-refractory to DBS. Therefore, in a final attempt, the basolateral amygdala was targeted bilaterally. Despite great efforts in DBS programming, OCD symptoms remained unchanged and amygdaloid stimulation was discontinued eventually. Table 4 outlines baseline features and results of DBS in case 3 as evaluated with our assessment battery.

Case 4. A 31-year-old single man with 10 years of education and a positive family history for OCD presented with predominantly compulsive acts (ICD-10: F42.1). The patient displayed first symptoms at age 11 including compulsive checking of doors and windows, interpreting numbers and colors, repeatedly touching objects, the compulsion not to step on stains on the floor and calculating rituals. As a consequence of the increasing severity of his symptoms he was never able to take up a profession. Detailed assessment revealed no history of previous psychosis or personality disorder. Preceding pharmacological trials involved treatment with at least two SSRIs and augmented therapy with atypical antipsychotics, benzodiazepines, serotonin-norepinephrine reuptake inhibitors (SNRIs) and dopamine agonists. Moreover, he underwent electroconvulsive therapy with a total of 17 sessions, but remained severely affected and disabled. Medication upon admission to our department included quetiapine (400 mg/d), duloxetine (60 mg/d) and paliperidone (6 mg/d).

**Fig 5:**
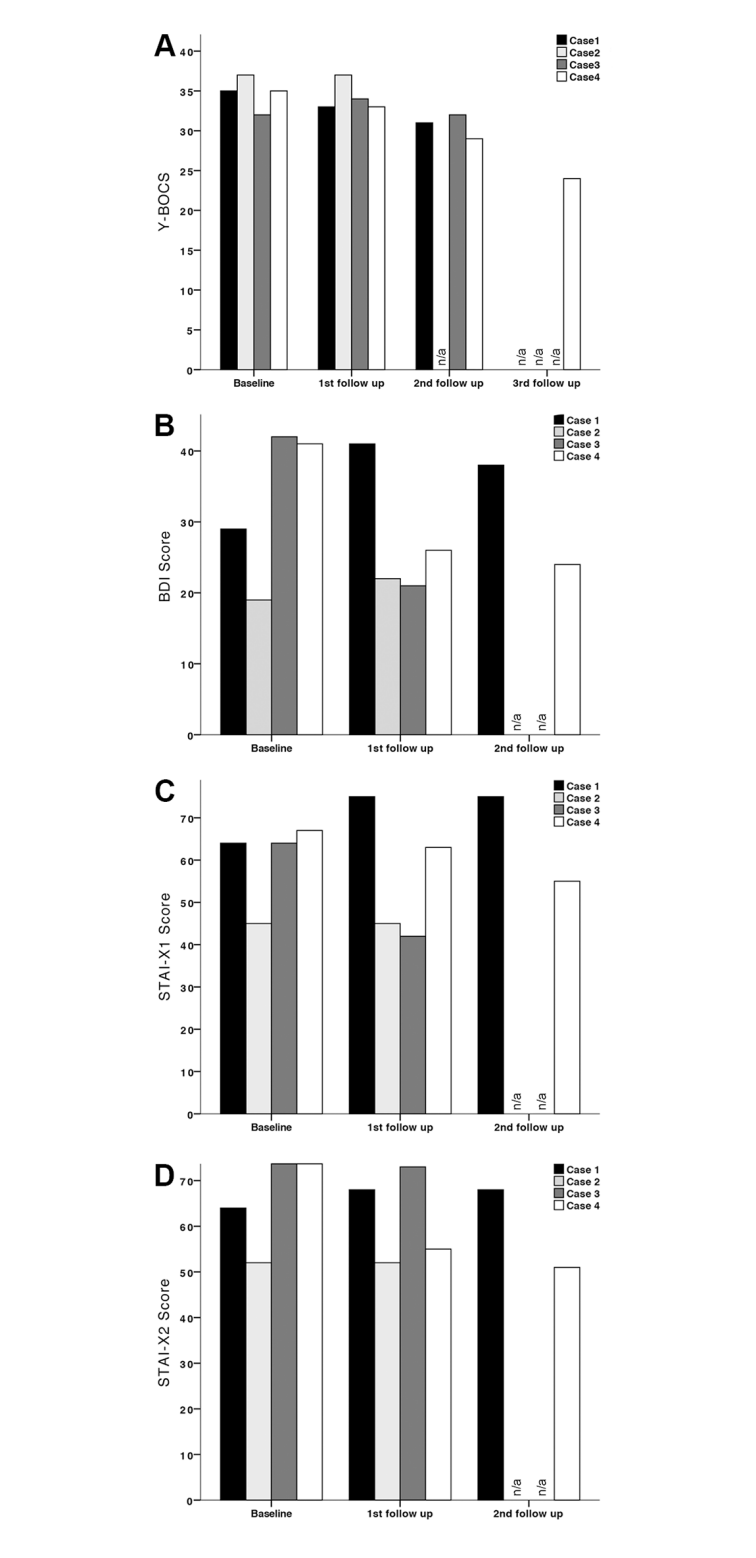
Bar graphs showing the efficacy of MD/VA stimulation. (A) Y-BOCS. (B) BDI. (C) STAI-X1. (D) STAI-X2. Scores of cases 1, 2, 3 and 4 are shown at presurgical baseline (Baseline) and following lead implantation (1st follow up, 2nd follow up, 3rd follow up). Intervals between follow up visits can be obtained from Table 4.

After lead placement into MD/VA, the patient initially displayed no improvement in symptom severity. As a consequence of the unsatisfying results and the patient’s discontent, stimulation parameters were adjusted and active contact points were varied. During follow up visits he reported persisting obsessions and compulsions as well as deterioration in mood. DBS was continued within the following years and stimulation parameters were adapted consistently. Interestingly, three years after lead placement, the OCD symptoms were found to be reduced (Fig 5A). The patient’s obsessive thoughts and compulsive acts had decreased by 31% (11 points) on the Y-BOCS scale.

### OCD symptom severity

According to the classification by Pallanti et al. [90] improvement in symptom severity of at least 35% on the Y-BOCS scale is defined as “full response”. In our study group none of the patients has reached that goal during the course of MD-DBS (Fig 5A). Only one of four patients (case 4) showed “partial response” as defined by symptom reduction between 25% and 35%, whereas three patients did not show any response to treatment with less than 25% improvement on the Y-BOCS. One patient (case 3) initially deteriorated during the observation period. No differential impact on compulsions and obsessions during MD stimulation could be determined.

### Depressive and anxiety symptoms

Depressive symptoms as assessed with the Beck Depression Inventory (BDI) (Fig 5B) dropped within the group receiving de novo lead implantation (case 3: -50%; case 4: -42%), whereas the rescue DBS group displayed symptom worsening (case 1: +31%; case 2: +16%) during MD-DBS. Anxiety symptoms were evaluated using the State-Trait Anxiety Inventory (Table 4), STAI-X1 for State anxiety (i.e. anxiety about an event) and STAI-X2 for Trait anxiety (i.e. anxiety as a personal characteristic). Scores improved in case 3 (STAI-X1: -34%; STAI-X2: -1%) and case 4 (STAI-X1: -17%; STAI-X2: -33%); in case 2, symptom severity remained unchanged, whereas case 1 displayed deterioration (STAI-X1: +17%; STAI-X2: +6%) at final follow-up evaluation (Fig 5C and 5D).

### Global functioning and quality of life

Psychological, occupational and social functioning as measured using the Global Assessment of Functioning (GAF) Scale remained stable in all patients (Table 4). Case 4 showed the most significant improvement in terms of quality of life with an 80% increase in MSQoL scores compared to baseline conditions. Case 2 did not show any changes in quality of life, case 1 (MSQoL: -28%) and case 3 (MSQoL: -19%) deteriorated during MD-stimulation.

### Neuropsychological assessment

Assessment of executive functioning was conducted using the Tower of London test (ToL)—number of right answers (Table 4): In cases 2 and 3 results were obtained at baseline and during MD-DBS, showing amelioration in case 2 (ToL: +25%) and deterioration in case 3 (ToL: -8%). Cases 1 and 4 were not assessed at baseline, though testing was performed throughout MD stimulation. Results showed an improvement in executive functioning in case 4 within the first year, whereas scores dropped in case 1 during the first 4 months of continuous stimulation. Verbal fluency was determined using the Verbal Fluency Examination (VFE), a phonemic test challenging the patient to name as many words as possible beginning with a certain initial letter within one minute (Table 4). While cases 2 and 3 showed no improvement during the observation period, the naming ability of case 4 ameliorated within one year of MD-DBS. In case 1, baseline characteristics had not been acquired, however, an improvement in verbal fluency could be noted during follow-up visits within the first 3 months of thalamic stimulation.

## Discussion

Among the studies reporting lead implantation into thalamic areas, the ithp has been targeted most commonly [28–30]. References favoring the MD/VA are sparse. Nuttin et al. published the only report on DBS in MD describing bilateral lead implantation in a rescue DBS attempt in one patient suffering from OCD. Postoperative evaluation revealed moderate response. In the long-term the patient did not benefit from MD -DBS [13]. The data obtained from our case series is in accordance with the observation of Nuttin et al. revealing no significant benefit of MD/VA stimulation on OCD symptom severity. Only case 4 responded to treatment, the benefit of MD/VA DBS in this patient, however, was moderate. Assessment of comorbid symptoms revealed a distinct improvement of depression and anxiety in the de novo DBS group. In cases 3 and 4 BDI scores dropped up to 50% whereas state and trait anxiety decreased by up to 30% (Table 4). To correlate the clinical findings with electrode positioning, backward calculation of lead localization was performed from stereotactic 2D X-ray and postoperative CCT images, stereotactic coordinates were then converted to the Atlas of the Human Brain [86] (Fig 4). Post-hoc analysis showed a correlation between alleviation of comorbid symptoms and more medial and ventral targeting of MD at the border and within MDMC, respectively. While medial lead placement seems to favor more extensive stimulation of MDMC, ventral targeting might allow stimulation of both cell bodies in close vicinity to the electrode and subthalamic fibers ascending to their respective cell bodies within MDMC. As amygdaloid afferents branch out within MDMC forming interlocking patches that claim a vast area, stimulation of the ventral aspect might lead to more successful recruitment of fibers implicated in the limbic and paralimbic circuit [61,91]. Adequate targeting of critical network connections as observed in the de novo group might consequently translate into better clinical outcome (Table 4). Target evaluation in the rescue DBS group revealed electrode localization predominantly in MDPC, VA, iml and mt (Fig 4); Contacts C1-1 and C2-1 (Fig 4) were found to be located at the border between MDMC and MDPC (Fig 4A). Stimulation of these contacts, however, did not lead to improvement of obsessive-compulsive and comorbid symptoms in the rescue DBS group, raising the question as to why stimulation in close vicinity to MDMC did not yield comparable clinical results in cases 1 and 2. Discrepancies might arise from intersubject neuroanatomical variability, that is difficult to account for due to technical limitations: Reference coordinates were obtained from the Atlas of the Human Brain and adjusted during treatment planning to match the patients’ individual neuroanatomy. This, however, only allows a rough estimation of the desired target point and necessitates further adaption and customization of the trajectory according to the present neuroanatomical findings. A draw back in targeting thalamic nuclei and subnuclei is the lack of anatomical information attainable from MR sequences, ruling direct targeting impossible. Hence, target determination can only be performed using indirect targeting, which, however, is prone to error and might not be adequate. Reliance on physiological markers as obtained from microelectrode recordings and/or intraoperative stimulation testing might have been a valuable adjunct in this study as they might have provided comprehensible functional feedback and circumvented the drawbacks concerned with imaging/indirect targeting. Post-hoc back calculation of lead location introduces potential error as well. Refractoriness to NA/IC DBS in the rescue DBS group is another confounding factor that needs to be addressed. It suggests an overall complex clinical picture in both cases, that did not respond to conventional DBS. Interpretation of outcome measures in this group thus proves especially difficult.

Within the rescue DBS group, both simultaneous/multifocal and staged stimulation attempts were performed to validate the therapeutic effect of DBS on OCD symptom severity. Moreover, a staged procedure was performed in case 3 due to refractoriness to MD/VA DBS. Superiority of simultaneous/multifocal stimulation over staged stimulation could not be observed in any of the patients, however we are skeptical of abandoning the multifocal approach: Psychiatric conditions are based on complex pathophysiological mechanisms that involve multiple, segregated neural circuits [42–46] that are affected to varying degrees in various patients and lead to different OCD symptom subtypes e.g. compulsive checking, hoarding or washing [92]. To achieve the best possible outcome, the therapeutic approach therefore requires tailored, symptom dependent targeting. Multifocal DBS might lead to more rational treatment in the future by enabling the determination of optimal DBS targets in complex and medically refractory cases and improve the efficacy of initial DBS treatment while minimizing the need for rescue DBS procedures in the long-term.

In our patient series, duration of thalamic stimulation varied between 4 and 35 months, raising the question whether stimulation duration has an influence on patient outcome. Among patients, short- and long-term stimulation did not show any significant improvement in OCD symptom severity. Case 4, however, who received thalamic stimulation for almost 3 years, displayed subtle alleviation of symptoms towards the end of the follow-up period. These rather unexpected results might arise from a variety of time related factors including continuous adjustment of stimulation parameters and long-term plastic changes in neural circuitry [42]. Following initial DBS programming in the immediate postoperative course, fine tuning of stimulation settings might take several months [93]. On the one hand, this is owing to the multitude of potential electrode configurations and stimulation parameters, which have to be adjusted consistently and individually. On the other hand, medical conditions and symptoms vary in clinical response to DBS and feature different latencies. In Parkinson’s disease, alleviation of rigidity and tremor can be observed within seconds of DBS, while response to bradykinesia has a latency of seconds to minutes [94]. Parkinsonian gait and balance tend to respond 20 to 30 minutes after stimulation onset. In contrast, symptoms in dystonia, depression and OCD are persevering and only decrease after months of continuous stimulation [10,95–97]. These findings suggest different mechanisms of action of DBS on neurons and circuitry in the short- and long-term. Long latencies are considered to be the result of neuroplastic and anatomical changes such as synaptic reorganization [98,99] whereas short-term improvements are likely induced by electrophysiological changes within the circuit [42]. Patients deciding to undergo DBS often experience great emotional pressure, since this form of therapy constitutes a treatment of last resort. Therefore, patients may easily lose confidence in the procedure if symptoms persist and may demand lead replacement or lead removal although the full potential of DBS has not developed. Hence, it lies within the responsibility of the treating physician to inform the patient about latencies in response to DBS. In case of prolonged stimulation failure, treatment response ought to be reevaluated in interdisciplinary DBS teams to confirm lead positioning and response to thresholds and exclude deterioration through psychiatric comorbidities or distress. Due to the growing number of DBS procedures, the need for reliable clinical predictors of outcome in DBS for therapy refractory OCD is increasing. Even in extensively explored targets DBS-treatment response only yields a 45% alleviation of OCD symptom severity on the Y-BOCS [100]. Thus, predictors of success need to be established in order to make an accurate statement about the indications for lead placement, lead replacement, lead removal and additional lead implantation.

Limitations in this study include the relatively small number of patients enrolled and the lack of control conditions. Furthermore, cases 1 and 3 exhibited distinct comorbidities on axis II of DSM-IV (Table 1) that, aside from potential inefficacy due to target selection, might have accounted for the failure of OCD symptom reduction using MD/VA DBS in these patients. Technical drawbacks concern the reliance on imaging modalities only. Hence, targeting 1) may not be accurate due to interindividual neuroanatomical variability, 2) may inadequately target the network connections as described above, and 3) as a consequence may not be accurately reflected in the post-hoc determination of electrode location.

Given the results of our small case series, yielding only a partial response in one patient, the overall strategy in targeting MD/VA as described in this paper cannot be recommended for DBS in therapy-refractory OCD. MDMC, namely the ventromedial portion may be a possible target in the treatment of mood related disorders such as major depressive disorder (MDD) and anxiety disorder, however, further research is necessary to make a clear statement about stimulation efficacy in using this target. We advise a cautious approach towards the use of multifocal and rescue DBS. Studies addressing these topics are sparse and guidelines regarding optimal management have yet to be established. Given the current lack of international patient registries that are in process of planning but not yet available, we feel compelled to provide this ‘negative’ retrospective trial to the scientific community.

## Supporting Information

S1 Appendix Statement of the Ethics Committee. (PDF)
